# Differential responses of macroinvertebrate ionomes across experimental N:P gradients in detritus-based headwater streams

**DOI:** 10.1007/s00442-020-04720-x

**Published:** 2020-08-01

**Authors:** Clay Prater, Phillip M. Bumpers, Lee M. Demi, Amy D. Rosemond, Punidan D. Jeyasingh

**Affiliations:** 1 Department of Geography, Loughborough University, Loughborough, UK; 2 Odum School of Ecology, University of Georgia, Athens, GA USA; 3 Department of Biology, University of Alabama, Tuscaloosa, AL USA; 4 Department of Integrative Biology, Oklahoma State University, Stillwater, OK USA

**Keywords:** Ecological stoichiometry, Nutrient enrichment, Benthos, Coarse benthic organic matter (CBOM)

## Abstract

Diverse global change processes are reshaping the biogeochemistry of stream ecosystems. Nutrient enrichment is a common stressor that can modify flows of biologically important elements such as carbon (C), nitrogen (N), and phosphorus (P) through stream foodwebs by altering the stoichiometric composition of stream organisms. However, enrichment effects on concentrations of other important essential and trace elements in stream taxa are less understood. We investigated shifts in macroinvertebrate ionomes in response to changes in coarse benthic organic matter (CBOM) stoichiometry following N and P enrichment of five detritus-based headwater streams. Concentrations of most elements (17/19) differed among three insect genera (*Maccaffertium sp.*, *Pycnopsyche spp.*, and *Tallaperla spp.*) prior to enrichment. Genus-specific changes in the body content of: P, magnesium, and sodium (Na) in *Tallaperla*; P, Na, and cadmium in *Pycnopsyche;* and P in *Maccaffertium* were also found across CBOM N:P gradients. These elements increased in *Tallaperla* but decreased in the other two taxa due to growth dilution at larger body sizes. Multivariate elemental differences were found across all taxa, and ionome-wide shifts with dietary N and P enrichment were also observed in *Tallaperla* and *Pycnopsyche*. Our results show that macroinvertebrates exhibit distinct differences in elemental composition beyond C, N, and P and that the ionomic composition of common stream taxa can vary with body size and N and P enrichment. Thus, bottom-up changes in N and P supplies could potentially influence the cycling of lesser studied biologically essential elements in aquatic environments by altering their relative proportions in animal tissues.

**Electronic supplementary material:**

The online version of this article (10.1007/s00442-020-04720-x) contains supplementary material, which is available to authorized users.

## Introduction

The biogeochemistry of stream ecosystems is currently being altered in myriad ways, including increases in alkalinity, heavy metals, and nutrients (Ferreira et al. 2016; Kaushal et al. 2018; Wurtsbaugh et al. 2019). Elevated concentrations of nitrogen (N) and phosphorus (P) are arguably the most ubiquitous changes, affecting a large proportion of global rivers and streams (USEPA 2016; UNEP 2019). Nutrient loading can impact streams by altering the relative proportions of biologically essential elements in producer and consumer tissues and modifying their flows through aquatic foodwebs (Cross et al. 2003; Singer and Battin 2007; Evans-White et al. 2009; Morse et al. 2012; Johnson et al. 2013). Further, these changes can also influence a variety of ecosystem functions including primary production, secondary production, decomposition rate, and whole-stream metabolism (Cross et al. 2006; Scott et al. 2008; Woodward et al. 2012; Dodds and Smith 2016; Demi et al. 2018; Kominoski et al. 2018). As these responses are collectively governed by bottom-up changes in elemental mass balance, it is critical to understand how N and P enrichment affects both organismal and ecosystem-level processes.

Significant progress towards this goal has been made by using the theory of ecological stoichiometry to study the effects of nutrient availability on stream ecosystems. Here, we focus on detritus-based streams where increased dissolved N and P inputs can stimulate microbial production (Gulis and Suberkropp 2003; Baldy et al. 2007; Suberkropp et al. 2010) and immobilization of these nutrients typically decreases detrital C:N and C:P ratios (Cross et al. 2003; Webster et al. 2009; Scott et al. 2013; Danger et al. 2016). This microbial conditioning reduces stoichiometric N and P imbalances between detrital basal food resources and detritivorous macroinvertebrates, providing higher quality food resources to consumers (Frost et al. 2002; Cross et al. 2005). Once assimilated, dietary N and P are used to build key biomolecules such as proteins and ribonucleic acids (Elser et al. 1996) that are in-turn used to construct new macroinvertebrate biomass, sometimes resulting in altered consumer body stoichiometry under excess nutrient supplies (Cross et al. 2003; Small and Pringle 2010; Morse et al. 2012). These changes at lower trophic levels may further propagate up the food web by influencing the nutrition and production of top-level predators in headwater streams (Davis et al. 2010; Bumpers et al. 2015, 2017) suggesting that resource supply stoichiometry is a primary factor controlling flows of C, N, and P through detritus-based ecosystems (Cross et al. 2007; Benstead et al. 2009). Overall, this body of research has revealed the intricate connections between environmental supply of macroelements, organismal metabolism, and ecosystem-level processes and highlights the need to consider these relationships when predicting ecological effects of stream nutrient enrichment.

Despite the substantial progress towards a mechanistic understanding of C, N and P cycling in headwater stream ecosystems, N and P enrichment effects on dynamics of the other ~ 20 inorganic elements that are necessary to sustain life remain understudied. These elements, collectively referred to as the ionome (Salt et al. 2008) or elementome (Peñuelas et al. 2019), play key biological roles including: signal transduction [sodium (Na), potassium (K), and calcium (Ca)], enzyme function [iron (Fe), copper (Cu), zinc (Zn), and Ca], and structure (Si and Ca; Frausto da Silva and Williams 2001). Similarly to C, N, and P, they are also connected through complex metabolic networks and can exhibit system-wide proportional changes under differential elemental supplies (e.g., Fe or P; Baxter et al. 2008; Jeyasingh et al. 2017). It is important to stress that most ionomic elements are essential by definition and that they have been demonstrated to limit biological production in organisms ranging from single-celled bacteria up to large vertebrates, including humans (Watanabe et al. 1997; Frausto da Silva and Williams 2001; Soetan et al. 2010). Thus, although comprising a relatively minor proportion of total body mass, changes in these elements in response to nutrient enrichment could have disproportionately strong effects on organismal metabolism. Nevertheless, the ecological importance of relatively few ionomic elements has been explored until recently due to a predominant focus on the law of the minimum and its emphasis on single macronutrient limitation (Kaspari and Powers 2016).

Pioneering work examining the influence of altered nutrient supply on organismal ionomes began in model plant systems (Baxter et al. 2008), and these effects are increasingly being studied in metazoans. For example, N- and P-limitation may alter the composition of several elements including manganese (Mn), nickel (Ni), and zinc (Zn) in both marine cyanobacteria and freshwater bacterial isolates (Twining et al. 2010; Jeyasingh et al. 2017). Ionomic shifts have also been detected in the aquatic consumer *Daphnia* (Jeyasingh et al. 2020) and in terrestrial weevil larvae (Ji et al. 2017) grown across dietary P gradients. When N and P supplies are sufficient, Na enrichment can increase insect abundance and diversity in tallgrass prairies (Prather et al. 2018). Ecologically relevant variation can also be found at higher trophic levels as ionomic differences have been documented across species and ontogenic stages of salamanders (Prater et al. 2019) and in threespine sticklebacks where morphological and ionomic adaptation to marine vs. freshwater environments can influence fish elemental uptake and excretion (Rudman et al. 2019). Like early stoichiometric work, these studies separately demonstrate correlations among environmental elemental supplies, consumer elemental composition, phylogeny/taxonomy, and phenotypic traits. However, our understanding of how consumer ionomic responses to dietary food quality influence higher-order ecological dynamics remains limited. To this end, taxon-specific differences in consumer ionomes under variable nutrient supplies can be explored to help advance our multi-elemental view of consumer ecology.

Here, we examined the effects of stream nutrient enrichment on the ionomes of three macroinvertebrate taxa: *Maccaffertium sp.* (Order Ephemeroptera), *Pycnopsyche spp.* (Tricoptera), and *Tallaperla spp.* (Plecoptera) using animals collected during a dissolved N and P addition experiment (Rosemond et al. 2015). Based on empirical tests of stoichiometric theory, we expected macroinvertebrate ionomes to differ with nutrient enrichment (hypothesis H1) but that these effects would largely depend on taxonomy (H2; Cross et al. 2003; Evans-White et al. 2005; González et al. 2018) and organismal body size (H3; Back and King 2013). We tested predictions that study organisms would show neutral or positive relationships between dietary and macroinvertebrate body N and P content and that these responses would be correlated with genus-specific ionomic changes. We also tested predictions that macroinvertebrate N, P, and other elements (excluding C) would be negatively related to body size (i.e., through growth dilution). Finally, we quantified the extent of ionomic differences among taxa and explored the potential for macroinvertebrate ionomic shifts in response to N and P enrichment to influence higher-order ecological processes by altering the production and community composition of these taxa in study streams.

## Methods

### Study sites, nutrient enrichment, and sample collection

We collected coarse benthic organic matter (CBOM) samples and macroinvertebrates as part of an experimental five-stream manipulative study conducted at the U.S.D.A Forest Service Coweeta Hydrological Laboratory, which serves as a long-term ecological research site in southwestern North Carolina, US. Study streams were located in the Dryman Fork watershed, which is a heavily forested and low-nutrient catchment (Rosemond et al. 2015). All streams shared similar topography, riparian vegetation, and physio-chemical properties and did not differ in macroinvertebrate abundance or biomass pre-enrichment (Bumpers et al. 2015; Manning et al. 2016; Demi et al. 2018).

Stream enrichment methods in these sites have been detailed extensively (Bumpers et al. 2015; Rosemond et al. 2015), but briefly, data for the current study were collected during one pre-enrichment year (July 2010–2011) and during 1 year of experimental dissolved N and P additions (July 2011–2012). We manipulated stream N:P ratios by dripping dissolved N (NH_4_NO_3_) and P (H_3_PO_4_) into 70-m stream reaches at discharge-weighted concentrations. Dissolved molar N:P target ratios of 2:1, 8:1, 16:1, 32:1, and 128:1 were achieved by covarying both N (81–650 μg L^−1^) and P (90–11 μg L^−1^) concentrations. We collected leaf litter CBOM at monthly intervals from each stream (Demi et al. 2018). Stream CBOM was collected across the entire wetted channel width from eight randomly selected 0.15 m transects, transported back to the laboratory at 4 °C, and dried at 60 °C for at least 24 h. We collected macroinvertebrates randomly during the first year of enrichment and 9 months post enrichment either by hand or D-frame net. Fresh specimens were sorted in the field, returned to the laboratory, and allowed to clear their guts for 24 h. Then, they were identified to genus and measured (total body length) to the nearest mm under at least 10 × magnification on a stereoscopic microscope affixed with a graduated stage. Animals were separated by size-classes, placed into trace-clean polyethylene tubes, and frozen. Frozen animals were later lyophilized for a minimum of 24 h.

We used mean annual CBOM N:P as our metric of nutrient enrichment to integrate temporal variation in resource quality over the course of pre and post-enrichment periods. We focused on leaf material N:P rather than dissolved nutrients because macroinvertebrates are known to obtain N and P from their diets rather than from dissolved uptake and detrital material represented the primary dietary material flows to all study taxa across both years (Demi et al. 2020; Supplementary Table 1). While all study taxa are primarily detritivores, it is important to note that there were smaller portions of other food resource items in the guts of animals collected alongside our study specimens (Demi et al. 2020). Similarly, there are also differences in functional feeding groups among these taxa as the *Pycnopsyche* and *Tallaperla* are classified as shredding macroinvertebrates and *Maccaffertium* are classified as scrapers and facultative collector-gatherers (Merritt et al. 2008). As discussed below, these factors are likely related to taxonomic differences in macroinvertebrate ionomes.

### Sample analyses

We ground CBOM samples into powder and measured N content of subsamples using an elemental analyzer (Carlo Erba NA 1500; Milan, Italy). We measured subsample P content by combustion (500 °C) and acid digestion of CBOM material followed by standard colorimetric/spectrophotometric P analysis (Allen 1974; APHA 1992). To minimize ontogenic effects on macroinvertebrate elemental profiles and to generate complete profiles on single individuals when possible (~ 50% of all measurements for *Pycnopsyche* and *Tallaperla*), we conducted elemental analyses on the largest specimens available for each taxon. All other measurements were conducted on composite tissues from 2–3 organisms from the same 1 mm size class.

Before conducting elemental analyses, we homogenized macroinvertebrate tissues in separate tubes using a motorized pestle. We then measured the C and N content from a subsample of each tube using a vario MICRO cube analyzer (Elemental Americas Inc., Mt. Laurel, NJ). Separate subsamples were digested using trace a 2:1 *v/v* solution of trace-metal grade nitric acid and hydrogen peroxide for a minimum of 24 h or until all tissues were completely dissolved. Following digestion, we diluted each sample with 10 ml of trace metal grade water and measured elemental profiles through inductively coupled plasma optical emission spectrometry (ICP-OES; Thermo Scientific iCAP 7400, Waltham, MA). All elemental concentrations were then divided by the subsample mass to express elemental composition as percentages. In all, we generated 80 ionomic profiles from taxa representing 3 common aquatic insect orders: *Maccaffertium* (*N* = 22), *Tallaperla* (*N* = 30), and *Pycnopsyche* (*N* = 28). These profiles consisted of estimates of 19 total elements: aluminum (Al), barium (Ba), C, Ca, cadmium (Cd), cobalt (Co), Cu, Fe, potassium (K), lithium (Li), magnesium (Mg), Mn, N, Na, P, sulfur (S), Si, strontium (Sr), and Zn. Limits of detection for ICP analyses are reported in Supplementary Table 2.

*Statistical Analyses:* To test the hypothesized effects of dietary N:P enrichment (H1), taxonomy (H2), and body size (H3) on macroinvertebrate elemental composition, we conducted a series of complementary analyses. Beginning with univariate tests, we contrasted elemental differences among genera (H2) using data from *pre-enrichment* animals only. Then, we examined genus-specific responses to dietary N:P enrichment (H1) and the influence of body size (H3) on organismal elemental composition using both *pre and post-enrichment* animals combined. Finally, we demonstrated how both of these factors were related to full ionomic changes in multivariate space using combined *pre and post-enrichment* datasets.

Prior to running parametric statistics, all elemental percentages were log transformed to better meet normality and variance assumptions (Goos et al. 2017; Prater et al. 2019). To compare taxonomic differences in elemental composition prior to enrichment, we first ran a one-way analysis of variance (ANOVA) followed by post hoc least squares mean t-tests among genera with significant differences determined by *P* values that were Bonferroni corrected for multiple comparisons (*P* < 0.05/3 = 0.017). We also used this procedure to test for differences in macroinvertebrate body size between pre-enrichment and enrichment years for each genus.

To contrast the relative influence of taxonomic differences and CBOM N:P enrichment on invertebrate elemental composition, we ran separate univariate mixed effects models for each element including the fixed effects *g* for genus, *e* for CBOM N:P, and *g* × *e* for genus × CBOM N:P interactions using combined pre and post-enrichment data. We also included body size *b* and genus × body size interactions *g* × *b* as fixed effects to test and control for effects of our intentional specimen size-selection procedure. Stream *s* was added as a random effect to account for non-independence of errors between elemental measurements on genera collected from the same stream, yielding a final model equation of:

1 $$y\, = \,g\, + \,e\, + \,b\, + \,\left( {g\, \times \,e} \right)\, + \,\left( {g\, \times \,b} \right)\, + \,s$$

This model was retained for all elements showing significant *b* or *g* × *b* effects, but was simplified to:

2 $$y\, = \,g\, + \,e\, + \,\left( {g\, \times \,e} \right)\, + \,s$$

using backward selection for elements unaffected by body size according to Zuur et al. (2009). For elements showing significant *g* × *e* effects after Bonferroni correction, we quantified genus-specific responses to food quality of each taxon across CBOM N:P gradients using the mixed effect model:

3 $$y\, = \,e\, + \,b\, + \,s$$

removing the term *b* when insignificant. Please note that, while all statistics were conducted on transformed data, untransformed elemental concentrations are reported in tables and figures for ease of interpretation and to facilitate comparisons across studies.

To examine multivariate changes in consumer ionomic profiles, we conducted principal components analysis (PCA) on combined pre and post-enrichment datasets. This analysis was performed using a correlational matrix, which standardizes variables to a mean of zero and a standard deviation of one to control for large differences in elemental concentration and variation within the dataset (Quinn and Keough 2002). We selected the optimal number of PCA components to retain using a broken-stick method (Jackson 1993). Eigen vector arrows depict the relative strength of relationships between each element and principle component. To facilitate visual interpretation of macroinvertebrate ionomes, PCA scores (i.e., individual points for each specimen measured shown in Euclidian space) were standardized by the square root of their eigen values (Oksanen et al. 2019).

To complement these ordinations, we first tested for ionomic differences among genera by comparing their PC scores while controlling for random stream effects using a mixed linear model. Then, we examined genus-specific ionomic changes across food quality gradients using Eq. 3. Finally, we contrasted variation in ionomic profiles vs. stoichiometric ratios among taxa using percentage differences of mean PCA centroids and mean C:N, C:P, and N:P ratios for each taxon. All statistics were conducted in R (version 3.5.3) using the package lme4 (Bates et al. 2015) for mixed models and vegan for PCA (Oksanen et al. 2019).

## Results

There were significant taxonomic differences in elemental composition for all but two elements (Ca and P) prior to enrichment. *Tallaperla* and *Maccaffertium* had the highest body N content, and *Tallaperla* %C was higher than the other two taxa (Table 1). The relative proportions of other elements also differed with *Maccaffertium* having the highest body content of most elements including Cd, Cu, Fe, S, and Si whereas *Pycnopsyche* showed the highest proportions of Ba, K, and Mn.

**Table 1:** Variation in macroinvertebrate elemental composition prior to experimental nutrient enrichment

	Element	*Maccaffertium*		*Pycnopsyche*		*Tallaperla*	
		Mean	SD	Mean	SD	Mean	SD
Bulk (%)	C	47.80^b^	3.10	48.80^b^	2.13	**50.80**^**a**^	2.08
	N	9.48^a^	1.68	**7.97**^**b**^	0.95	9.76^a^	1.17
	P	0.945^a^	0.094	0.919^a^	0.140	0.980^a^	0.145
	S	**0.783**^**a**^	0.047	0.490^b^	0.074	0.449^b^	0.050
	K	0.684^b^	0.168	**1.974**^**a**^	0.795	0.541^b^	0.194
	Na	0.371^a^	0.110	0.426^a^	0.154	**0.209**^**b**^	0.124
	Ca	0.325^a^	0.114	0.369^a^	0.163	0.351^a^	0.187
	Mg	**0.126**^**b**^	0.042	0.197^a^	0.061	0.224^a^	0.092
Trace (μg mg^−1^)	Al	1.744^a^	0.716	0.791^a^	0.572	**0.557**^**b**^	0.647
	Fe	**1.055**^**a**^	0.475	0.443^b^	0.298	0.373^b^	0.422
	Zn	**0.302**^**b**^	0.181	0.528^a^	0.150	0.929^a^	0.678
	Si	**0.516**^**a**^	0.172	0.300^b^	0.182	0.198^b^	0.174
	Mn	**0.0963**^**b**^	0.0265	**0.904**^**a**^	0.432	**0.0491**^**c**^	0.0285
	Ba	0.0335^b^	0.0089	**0.770**^**a**^	0.370	0.0294^b^	0.0149
	Cu	**0.0380**^**a**^	0.0223	0.0185^b^	0.0047	0.0198^b^	0.0043
	Sr	**0.00916**^**b**^	0.00341	0.0216^a^	0.0069	0.0179^a^	0.0089
	Cd	**0.00691**^**a**^	0.00202	**0.00148**^**c**^	0.00069	**0.00042**^**b**^	0.00020
	Co	0.00489^a^	0.00348	0.00261^a^	0.00141	**0.00077**^**b**^	0.00034
	Li	0.00061^a^	0.00037	**0.00029**^**b**^	0.00017	0.00031^a^	0.00014

Significant taxonomic differences in mean elemental concentrations were determined using least squares mean *t*-tests where *P *values were adjusted using Bonferroni corrections (*P* = 0.017). Unique elemental concentrations among the three study taxa are indicated by bold font, and notational letters indicate concentration differences from higher^a^ to lower^bc^. Bulk element concentrations (> 0.1% dry mass) are reported in % dry mass, whereas trace elements (< 0.1%) are reported in μg mg^−1^. Genus sample sizes for pre-enrichment measurements are: *Maccaffertium* (*N* = 7), *Tallaperla* (*N* = 15), and *Pycnopsyche* (*N* = 14)

**Fig. 1:**
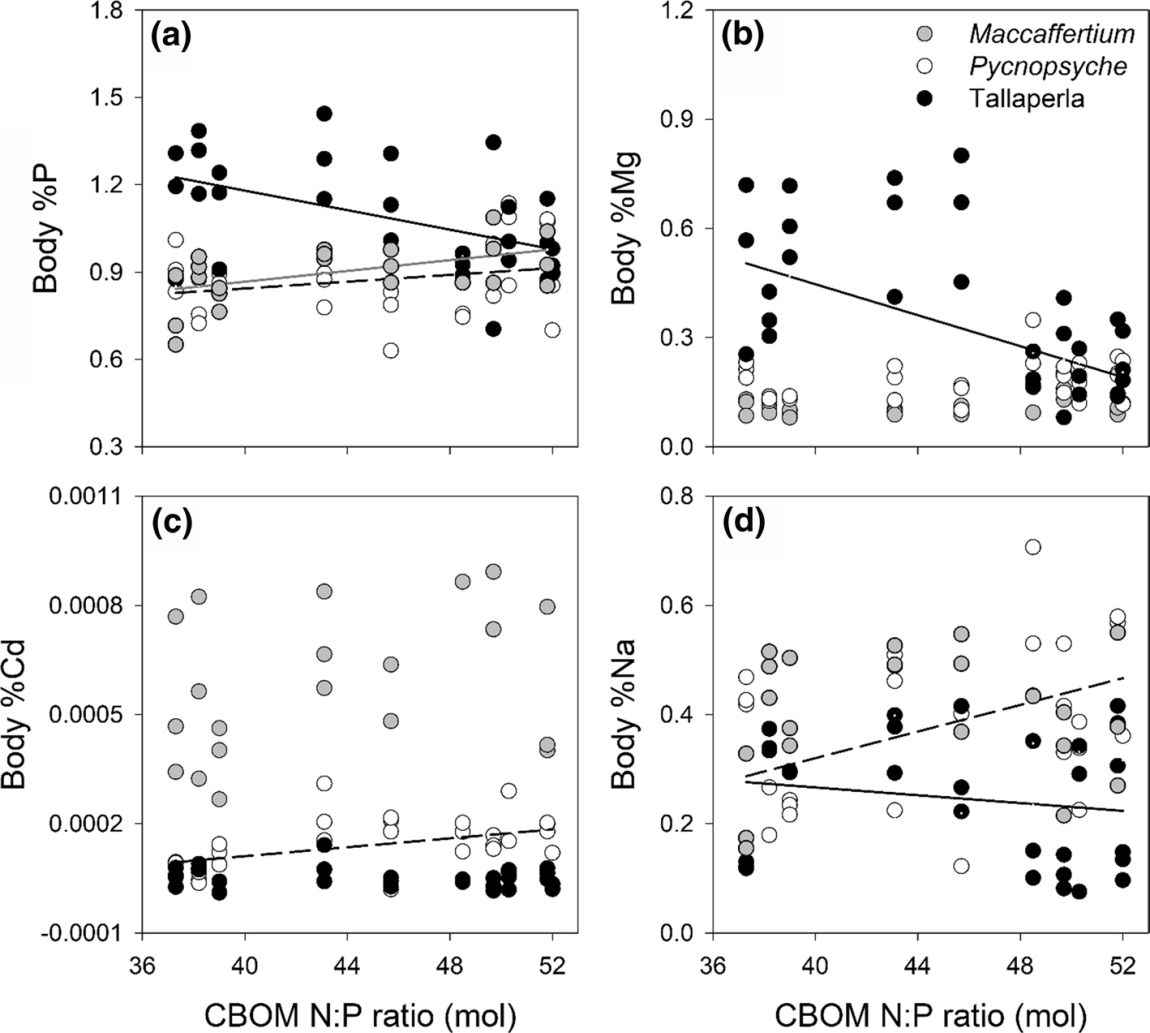
Taxonomic differences in body elemental composition across dietary resource stoichiometry gradients in pre and post-enrichment years combined. Significant relationships between macroinvertebrate body elemental composition and coarse benthic organic matter nitrogen:phosphorus ratios (CBOM N:P) are shown as regression lines fit using mixed effects models. Changes in: **a** Body %P were found for all taxa (*Maccaffertium*, *y* = 0.005*x*–0.260, *N* = 22; *Pycnopsyche*, *y* = 0.004*x*–0.253, *N* = 30; *Tallaperla*, y = − 0.007*x* + 0.337, *N* = 28), %Mg for *Tallaperla* (*y* = − 0.018*x* + 0.356), %Cd for *Maccaffertium* (*y* = 0.022*x*–4.878), and %Na for both *Pycnopsyche* (*y* = 0.013*x*–1.037) and *Tallaperla* (*y* = − 0.023*x* + 0.386). Regression lines are colored to match symbols for each genus (*Pycnopsyche* lines are dashed). All elements are reported in standard scientific notation

In addition to elemental differences among taxa, N and P enrichment and body size affected macroinvertebrate elemental composition. Body Ba and S content increased similarly across CBOM N:P gradients in all taxa (Table 2). However, there were also genus-specific responses to dietary N and P enrichment. *Tallaperla* showed decreased %P, Mg, and Na at higher food N:P ratios (Fig. 1). In contrast, *Pycnopsyche* body P, Cd, and Na content and *Maccaffertium* %P increased across these gradients. We were unable to identify any other univariate elemental changes due to high within-stream variance in macroinvertebrate elemental composition. Part of this variation could be explained by body size effects (Table 2), as concentrations of most elements were negatively related to body size in all taxa. However, %C increased in larger *Maccaffertium* and *Pycnopsyche* but stayed relatively consistent in *Tallaperla*. Body %P, Ca, Mg, and Zn increased with body size in *Tallaperla* while either decreasing (Ca and Mg) or not changing (Zn) in the other two taxa. It is important to note that body size was not related to nutrient enrichment, as body size did not differ between pre and post-enrichment years for any genus (*Maccaffertium*, *P* = 0.610; *Pycnopsyche, P* = 0.973; *Tallaperla, P* = 0.090).

**Table 2:** Differences in elemental content among genera and across resource stoichiometry gradients in pre and post-enrichment years combined

Factor	Element	SS	MS	*df* num	~ *df* den	*F*	*P* value
Genus	Al	1.277	0.638	2	70	7.10	**0.002**
	Ba	0.099	0.050	2	71	1.13	0.330
	Cd	0.331	0.165	2	70	3.59	0.033
	Co	0.329	0.165	2	71	4.98	**0.010**
	Fe	1.278	0.639	2	70	9.88	** < 0.001**
	Li	0.263	0.131	2	70	5.21	**0.008**
	Mg	0.027	0.014	2	71	0.74	0.479
	Na	0.150	0.075	2	70	2.68	0.076
	P	0.087	0.043	2	71	13.83	** < 0.001**
	S	0.005	0.002	2	72	0.72	0.489
	Si	0.760	0.380	2	73	7.35	**0.001**
CBOM N:P	Al	0.046	0.046	1	72	0.51	0.476
	Ba	0.369	0.369	1	59	8.36	**0.005**
	Cd	0.001	0.001	1	73	0.03	0.861
	Co	0.014	0.014	1	64	0.42	0.520
	Fe	0.031	0.031	1	72	0.47	0.494
	Li	0.002	0.002	1	73	0.07	0.790
	Mg	0.032	0.032	1	71	1.81	0.182
	Na	0.048	0.048	1	72	1.72	0.194
	P	0.001	0.001	1	74	0.21	0.650
	S	0.020	0.020	1	63	6.32	**0.015**
	Si	0.231	0.231	1	73	4.47	0.038
Genus × CBOM N:P	Al	0.466	0.231	2	71	2.60	0.082
	Ba	0.263	0.131	2	72	2.97	0.058
	Cd	0.477	0.238	2	70	5.17	**0.008**
	Co	0.129	0.064	2	71	1.95	0.150
	Fe	0.525	0.262	2	71	4.06	0.022
	Li	0.203	0.101	2	70	4.01	0.023
	Mg	0.272	0.136	2	71	7.62	**0.001**
	Na	0.325	0.163	2	70	5.86	**0.005**
	P	0.064	0.032	2	71	10.16	** < 0.001**
	S	0.018	0.009	2	72	2.91	0.061
	Si	0.341	0.170	2	73	3.30	0.043
Body size	Al	2.037	2.037	1	73	22.66	** < 0.001**
	Ba	0.363	0.363	1	72	8.21	**0.006**
	C	0.003	0.003	1	71	7.42	**0.008**
	Cd	0.502	0.502	1	71	10.91	**0.002**
	Co	0.495	0.495	1	73	14.97	** < 0.001**
	Fe	1.752	1.752	1	73	27.07	** < 0.001**
	Li	0.260	0.002	1	72	10.29	**0.002**
	Na	0.347	0.347	1	71	12.47	** < 0.001**
	Si	1.942	1.942	1	73	37.56	** < 0.001**
Genus × Body size	Ca	0.422	0.211	2	70	8.91	** < 0.001**
	Mg	0.571	0.285	2	71	15.99	** < 0.001**
	Zn	0.841	0.421	2	71	13.68	** < 0.001**

*SS* sum of squares, *MS* mean squares,* df num* numerator degrees of freedom, *~ df den* approximate denominator degrees of freedom, *F* F-ratio of mean squares, and *P* values are reported for mixed linear models. Significant differences are shown in bold using *P* values adjusted using Bonferroni corrections. Abbreviations include coarse benthic organic matter (CBOM) and nitrogen to phosphorus (N:P) ratio. Please note that certain elements are omitted from the table as no significant differences were found, and body size effects removed in the process of model selection are not shown

**Fig. 2:**
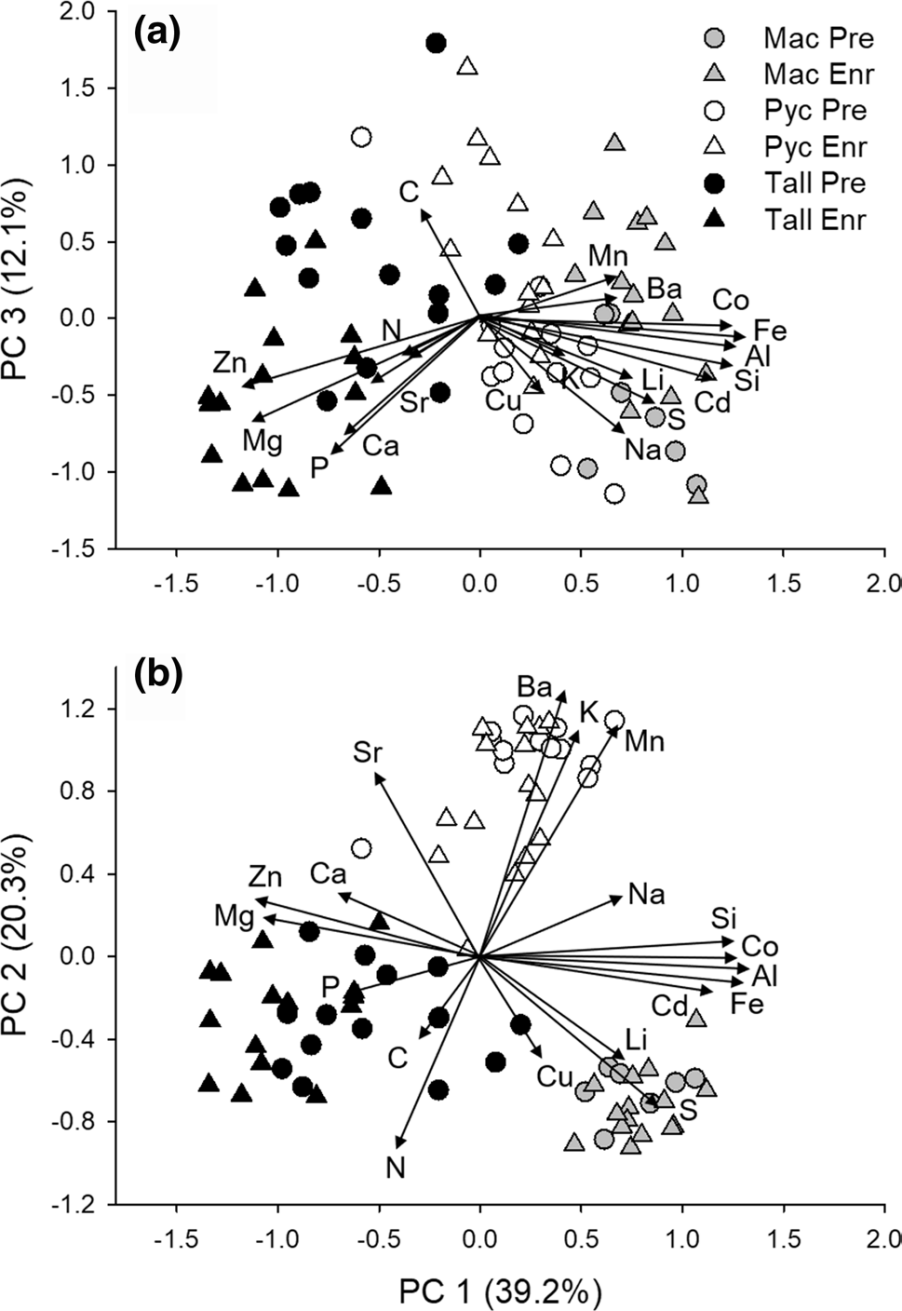
Multivariate differences in macroinvertebrate ionomic profiles in pre and post-enrichment years combined. The amount of variance explained by each principle component (PC) is reported for **a** PC1 vs. PC3 and **b** PC1 vs. PC2. Circles and triangles represent profiles from pre-enrichment (Pre) and post-enrichment (Enr) individuals for each genus, respectively. Elemental vectors represent the relative strength of correlations between individual elements and axes genus names are abbreviated as *Maccaffertium* (Mac), *Pycnopsyche* (Pyc), and *Tallaperla* (Tal)

**Fig. 3:**
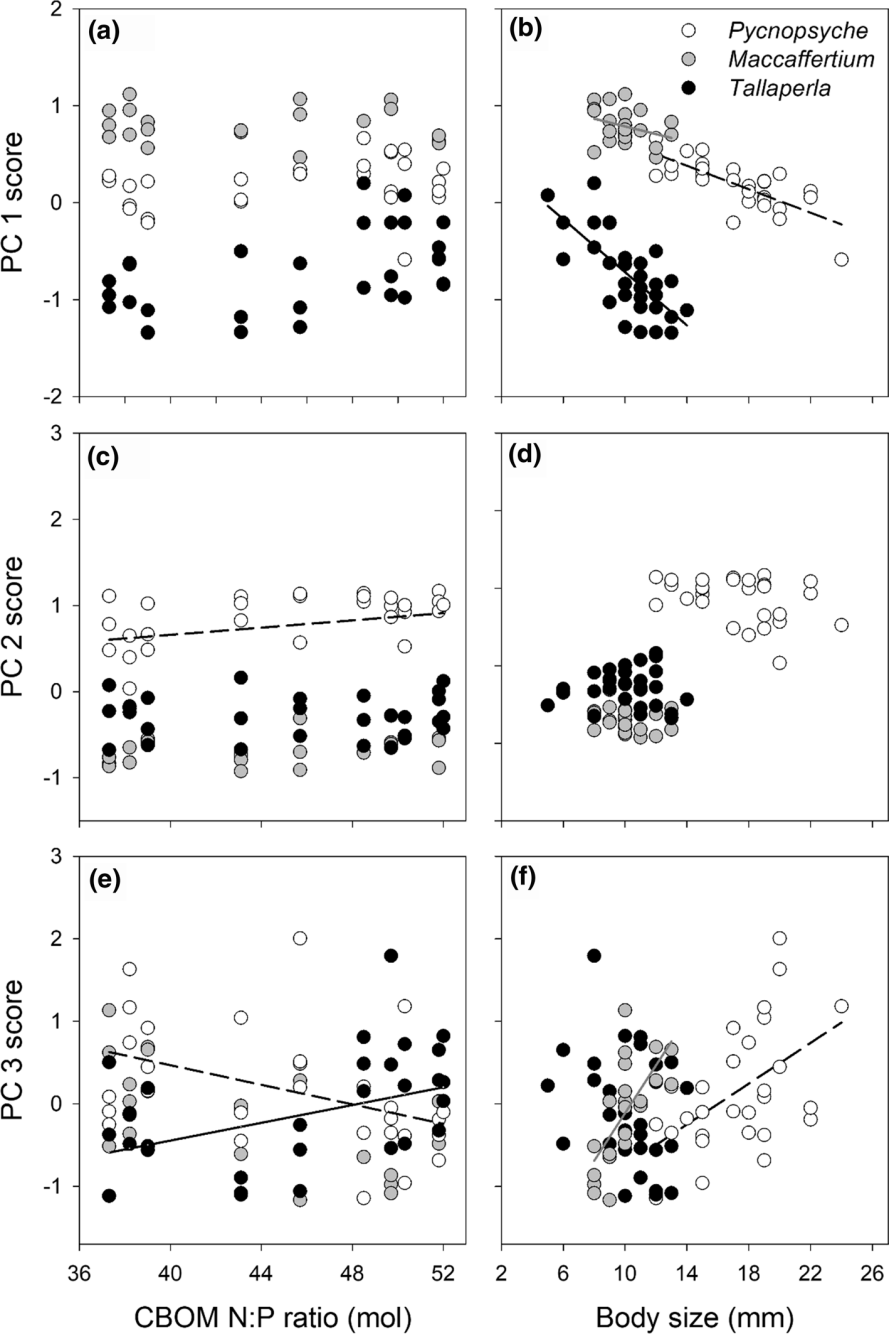
Effects of dietary nitrogen:phosphorus (N:P) enrichment on macroinvertebrate ionomic profiles in pre and post-enrichment years combined. Significant relationships between macroinvertebrate principle component (PC) loadings and CBOM N:P ratios (as abbreviated in Fig. 1) and between PC loadings and body size are shown as regression lines fit using mixed effects models. Changes in **c** PC2 loadings were found for *Pycnopsyche* (*y* = 0.025*x*–0.253) and **e** PC3 loadings for *Pycnopsyche* (y = -0.060x + 2.868) and *Tallaperla* (*y* = 0.060–2.830) across CBOM N:P gradients. Body size was related to differences in **b** PC1 loadings for all taxa (*Maccaffertium*, *y* = − 0.067*x* + 1.468; *Pycnopsyche*, *y* = − 0.060*x* + 1.220; *Tallaperla*, *y* = − 0.117*x* + 0.453) and **f** PC3 loadings for *Maccaffertium* (*y* = 0.141*x*–2.284) and *Pycnopsyche* (*y* = 0.236–2.473)

In addition to univariate responses, multivariate ionomic profiles also differed among genera and across dietary nutrient gradients. Principal components analysis explained 71.5% of macroinvertebrate ionomic variation with significant genus differences found across PC’s 1 and 2 (*P* < 0.001). *Tallaperla and Maccaffertium* profiles exhibited the most distinct elemental composition and separated out across PC1 (Fig. 2), while *Pycnopsyche* profiles separated out across PC2 and were positively related to CBOM N:P along this axis (Table 3; Fig. 3c). Macroinvertebrate ionomes showed a greater degree of overlap across PC3 but were differentially influenced by N and P enrichment as PC3 scores of *Pycnopsyche* decreased and *Tallaperla* scores increased across CBOM N:P gradients (Fig. 3e).

**Table 3:** Changes in macroinvertebrate ionomes across food resource stoichiometry gradients

Genus	PC	Factor	SS	MS	*df* num	~ *df* den	*F*	*P* value
*Maccaffertium*	PC1	CBOM N:P	0.068	0.068	1	17	2.70	0.119
		Body size	0.149	0.149	1	14	5.91	0.029
	PC2	CBOM N:P	0.044	0.044	1	15	2.08	0.170
	PC3	CBOM N:P	0.504	0.504	1	19	2.50	0.131
		Body size	2.109	2.109	1	19	10.45	**0.004**
*Pycnopsyche*	PC1	CBOM N:P	0.083	0.083	1	25	2.94	0.100
		Body size	0.933	0.933	1	25	32.89	** < 0.001**
	PC2	CBOM N:P	0.448	0.448	1	24	10.09	**0.004**
		Body size	0.175	0.175	1	24	3.93	0.059
	PC3	CBOM N:P	2.635	2.635	1	23	9.96	**0.004**
		Body size	4.459	4.459	1	24	16.86	** < 0.001**
*Tallaperla*	PC1	CBOM N:P	0.197	0.197	1	27	2.34	0.138
		Body size	1.548	1.548	1	27	18.32	** < 0.001**
	PC2	CBOM N:P	< 0.001	< 0.001	1	28	< 0.01	0.969
	PC3	CBOM N:P	3.210	3.210	1	26	8.68	**0.006**

Relationships between principle component (PC) loadings of each genus and CBOM N:P (as abbreviated in Table 2) were determined using mixed effects models. Body size was included as a model covariate when significant after Bonferroni correction (*P* = 0.025). All significant relationships are shown in bold

Body size also affected macroinvertebrate ionomes. Body size variation was strongly related to PC1, where loadings decreased with body size in all taxa (Table 3; Fig. 3b). No body size effects were found for PC2, but body size differentially affected ionomic profiles across PC3 as *Maccaffertium* and *Pycnopsyche* scores increased with body size, while *Tallaperla* scores did not change (Fig. 3f). Since body size and CBOM N:P effects on *Tallaperla* were orthogonal (i.e., affecting separate axes), body size did not overly influence their PC3 scores. Thus, *Tallaperla* ionomic changes along this axis are consistent with decreased body %P, Mg, and Na across CBOM N:P gradients reported above and were also associated with decreased Ca, Zn, Sr, and N content in this genus (Figs. 2a and 3e). Similar decreases in these elements along with lower S, Li, and Cu concentrations and higher Ba, K, and Mn content were indicated by changes in *Pycnopsyche* PC2 scores across CBOM N:P gradients (Figs. 2b and 3c). However, body size differences across PC3 masked nutrient enrichment effects along this axis for *Pycnopsyche* and likely explain our inability to detect CBOM N and P enrichment effects on *Maccaffertium* ionomes (Fig. 3f).

## Discussion

This study documented ionomic variation in three common macroinvertebrate detritivores collected from streams undergoing N and P enrichment. These genera differed in traditionally studied stoichiometric elements (i.e., C and N) in addition to 15 other elements prior to nutrient enrichment. There were also unique genus-specific changes in elemental composition, as *Tallaperla* and *Pycnopsyche* showed ionomic shifts across CBOM N:P gradients, while *Maccaffertium* elemental concentrations were weakly related to nutrient enrichment due to confounding effects of body size. These results extend previous observations of both taxonomic and body size mediated effects on consumer responses to dietary nutrient content (Cross et al. 2003; Evans-White et al. 2005; Karimi and Folt 2006; Benstead et al. 2014) suggesting that, in addition to N and P, stream nutrient enrichment could potentially alter the cycling of many other biologically essential elements in detritus-based ecosystems.

Macroinvertebrate elemental composition differed substantially across study taxa. While %P was similar among genera before enrichment, *Tallaperla* had the highest body C content out of the three and higher N content than *Maccaffertium*, confirming general patterns in macroinvertebrate stoichiometry documented in the region (Cross et al. 2003). Consistent with prior work (Karimi and Folt 2006), there were also taxonomic differences in multivariate macroinvertebrate ionomes that accounted for a considerable amount of elemental variation. Whole ionomic variation was far more extensive than stoichiometric C, N, and P variation across study taxa (Table 4), supporting the idea that multivariate elemental analyses provide a more detailed way for studying taxonomic differences in organismal elemental composition (Peñuelas et al. 2019; Prater et al. 2019). However, it should be noted that ionomic patterns are unlikely to be controlled strictly by taxonomy, as consumer elemental composition is a complex function of life-history trait evolution/expression, consumer nutritional physiology, and feeding behavior—processes themselves which are tied to environmental elemental supplies.

**Table 4:** Differences in multivariate ionomes and stoichiometric values among genera

(a)	PC1	PC2	PC3
	*Maccaffertium*	*Pycnopsyche*	*Tallaperla*
*Maccaffertium*	–	144%	4%
*Pycnopsyche*	41%	–	23%
*Tallaperla*	150%	90%	–

(b)	C:N	C:P	N:P
	*Pycnopsyche*	*Tallaperla*	*Maccaffertium*
*Pycnopsyche*	–	20%	18%
*Tallaperla*	21%	–	17%
*Maccaffertium*	19%	19%	–

Percent differences were calculated using mean principle component (PC) centroids and molar stoichiometric ratios for each taxon. Columns show contrasts with the most distinct taxon separating across each PC and for each stoichiometric ratio

In addition to taxonomy, macroinvertebrate elemental content also differed across resource stoichiometry gradients. *Tallaperla* body %P was negatively related to CBOM N:P and was higher than the other two groups after nutrient enrichment. Elevated *Tallaperla* body %P was accompanied by increased %Mg and %Na along with a suite of multivariate changes, illustrating the complex metabolic regulation of ionomes under nutrient enrichment (Baxter et al. 2008). In contrast, *Pycnopsyche* body P, Cd, and Na content and *Maccaffertium* body %P was positively related to CBOM N:P. Based on previous work, we expected to see either no change or negative relationships between macroinvertebrate body %P and detrital N:P for these taxa (Cross et al. 2003; Kendrick and Benstead 2013; Halvorson et al. 2019), making it initially difficult to reconcile the positive relationships found in our study. However, upon closer inspection these effects can largely be attributed to artifacts of stochastic body size variation within/among streams.

Body size-stoichiometry relationships are well established in animals (Elser et al. 1996; El-Sabaawi et al. 2012; Back and King 2013) but have only recently started to be explored at the ionomic level (Ma et al. 2015). Incorporating these relationships into ionomic frameworks is essential as they can strongly influence ecological interpretations from field-collected animals. For example, body size-ionome correlations can explain our contradictory findings of increases in *Pycnopsyche* body P and other correlated elements with CBOM P enrichment across PC2 but opposite patterns across PC3 (Fig. 3). Body size was not correlated to PC2, but it explained > 40% more variation than CBOM enrichment effects on PC3, effectively overriding and masking enrichment effects along this axis. Focusing on PC2, *Pycnopsyche* body %P changes are consistent with a priori predictions of positive relationships between dietary and organismal N:P, and ionomic responses are remarkably similar to those of *Tallaperla*. Positive relationships between *Pycnopsyche* body size and %C and decreases in all other elements across PC3 indicate that ionomic shifts along this axis most likely resulted from growth dilution in larger individuals. Similar patterns across aquatic nutrient gradients have been reported for several macroinvertebrate taxa (Karimi et al. 2010) and seem to also partially explain *Maccaffertium* ionomic shifts across PC3, highlighting the importance of accounting for body size effects in ionomic studies.

In addition to body size, it is likely that other biological factors also influenced macroinvertebrate elemental composition. Stream CBOM P enrichment did not lead to increased *Maccaffertium* P content unlike previous observations from the region (Cross et al. 2003). We did confirm higher P content (along with Ca, Mg, and Zn) in larger-sized *Tallaperla*, but these results are counter to consistent negative relationships between body size and %P reported across several macroinvertebrate orders (Back and King 2013). These inconsistencies along with extensive elemental variation in our study animals suggest that other factors such as sex (Back and King 2013; Goos et al. 2017) or nutrient storage (Bertram et al. 2008) could also play an important role in shaping ionomic composition in these populations. Variation in food preference (or simply stochastic resource availability) is also plausible for *Maccaffertium* because, while functioning predominately a detritivore in this study, its diatom consumption increased with P enrichment and higher algal biomass in our study streams (Bumpers et al. 2017; Demi et al. 2020). Diatom supplementation may further explain the high Si concentrations in this genus and potentially of other trace elements such as Co, Fe, Al and Cd as well. We cannot properly quantify the effects of dietary shifts within our dataset, but these results reinforce the idea that autotrophic diet supplementation under N and P enrichment can be an important factor affecting animal nutritional ecology in stream food webs (Brett et al. 2017; Crenier et al. 2017).

Higher-order ecological consequences of ionomic changes in consumers have yet to be systematically explored, but there are at least two potential pathways for these changes to occur in our study area. First, macroinvertebrate community production increased with stream N and P enrichment (Demi et al. 2018), but production did not change symmetrically across all taxa as *Tallaperla* and *Maccaffertium* production increased more on average than *Pycnopsyche* (Supplementary Table 3). As *Tallaperla* and *Maccaffertium* are the two most common prey items by biomass for an important salamander species in these streams (*Desmognathus quadramaculatus*), changes in their production and/or elemental composition under nutrient enrichment could feed back to influence nutrition and production of these vertebrate predators (Bumpers et al. 2017). Secondly, previous work has documented negative relationships between body K and Ca composition and excretion in four common taxa in Coweeta streams, including *Maccaffertium*, which suggests that taxonomic changes in macroinvertebrate communities may alter nutrient cycling in these streams in a stoichiometric manner (Webster and Patten 1979). While preliminary, these observations indicate that in addition to affecting individual elemental composition, N and P enrichment of basal food resources could also alter flows of other elements through detrital foodwebs by differentially affecting production (Demi et al. 2020) and/or excretion of individual genera with distinct elemental phenotypes (Rudman et al. 2019).

In this study, we documented taxonomic differences and changes in macroinvertebrate consumer ionomes across detrital N:P gradients. These observations build on previous stoichiometric work by showing how N and P enrichment can cause shifts in macroinvertebrate communities that could influence the dynamics of other non-typically studied elements in streams. As similar ionomic changes have been reported for many other plant, microbial, and consumer taxa (Baxter et al. 2008; Jeyasingh et al. 2017; Ji et al. 2017), they are likely to play an important role in the cycling of many biologically essential elements. Because of the inherent difficulties in precisely quantifying the ionomic composition of macroinvertebrate diets, we were unable to separate the effects of altered resource N:P from other elemental changes or to investigate the effects of proportional shifts in basal food resource biomass and ionomic composition on macroinvertebrate elemental composition. Since our work was exploratory and relied on specimens previously collected to address different objectives, experiments explicitly designed to manipulate and track flows of these elements through organisms and ecosystems are needed to fully appreciate the effects of N and P enrichment on stream foodwebs. This study adds to the growing body of work demonstrating the importance of extending our focus beyond traditionally studied elements (Jeyasingh et al. 2014, 2017; Kaspari and Powers 2016; Peñuelas et al. 2019) and suggests that, by broadening the scope of elemental ecology, we can gain a more complete understanding of how nutrient enrichment influences organismal metabolism and ultimately shapes ecosystem productivity.

## Electronic supplementary material

Below is the link to the electronic supplementary material.

Supplementary file1 (DOCX 30 kb)
