# Supplementary material for: Differential responses of macroinvertebrate ionomes across experimental N:P gradients in detritus-based headwater streams
Source: Oecologia. 2020 Aug 1;193(4):981–93. doi: 10.1007/s00442-020-04720-x (PMC7458898; doi:10.1007/s00442-020-04720-x)
Supplement: Supplementary file 1 — Supplementary file1 (DOCX 30 kb) [file 442_2020_4720_MOESM1_ESM.docx]

**Supplementary Information for:**

Differential responses of macroinvertebrate ionomes across experimental N:P gradients in detritus-based headwater streams

Clay Prater^1†^, Phillip M. Bumpers^2^, Lee M. Demi^3^, Amy D. Rosemond^2^, & Punidan D. Jeyasingh^4^

^1^Department of Geography, Loughborough University, Loughborough, UK

^2^ Odum School of Ecology, University of Georgia, Athens GA, USA

^3^ Department of Biology, University of Alabama, Tuscaloosa AL, USA

^4^ Department of Integrative Biology, Oklahoma State University, Stillwater OK, USA

^†^Contact information of corresponding author, mail to: [prater.clay@gmail.com](mailto:prater.clay@gmail.com)

**Supplementary Table 1.** Material Flows (g ash-free dry mass m^-2^ y^-1^) to primary consumer taxa reproduced from: Demi LM, Benstead JP, Rosemond AD, Maerz JC (2020) Experimental N and P additions relieve stoichiometric constraints on organic-matter flows through five stream food webs. J Anim Ecol 89:1468–1481. doi: 10.1111/1365-2656.13197

| Genus | Stream N:P | Enrichment | Amorphous Detritus | Animals | Diatoms | Fungi | Leaf Detritus | Wood |
| --- | --- | --- | --- | --- | --- | --- | --- | --- |
| *Maccaffertium sp.* | 8 | Pre | 0.6 | 0.0 | 0.0 | 0.1 | 0.8 | 0.0 |
|  |  | Post | 0.4 | 0.0 | 0.1 | 0.1 | 1.0 | 0.0 |
|  | 16 | Pre | 0.4 | 0.0 | 0.0 | 0.1 | 0.6 | 0.0 |
|  |  | Post | 0.6 | 0.0 | 1.0 | 0.1 | 0.7 | 0.7 |
|  | 32 | Pre | 0.8 | 0.0 | 0.0 | 0.0 | 0.3 | 0.0 |
|  |  | Post | 0.8 | 0.0 | 0.1 | 0.1 | 0.9 | 0.0 |
|  | 128 | Pre | 0.9 | 0.0 | 0.0 | 0.0 | 0.3 | 0.0 |
|  |  | Post | 0.2 | 0.0 | 0.3 | 0.0 | 0.5 | 0.0 |
| *Pycnopsyche spp.* | 2 | Pre | 0.5 | 0.0 | 0.0 | 0.4 | 3.8 | 0.2 |
|  |  | Post | 0.7 | 0.0 | 0.0 | 0.9 | 15.8 | 5.0 |
|  | 8 | Pre | 0.2 | 0.0 | 0.0 | 0.2 | 4.5 | 0.7 |
|  |  | Post | 0.1 | 0.0 | 0.0 | 0.3 | 5.7 | 3.4 |
|  | 16 | Pre | 0.6 | 0.0 | 0.0 | 0.2 | 4.4 | 0.6 |
|  |  | Post | 0.4 | 0.0 | 0.1 | 0.4 | 8.2 | 0.2 |
|  | 32 | Pre | 0.0 | 0.0 | 0.0 | 0.1 | 3.0 | 0.3 |
|  |  | Post | 0.2 | 0.0 | 0.0 | 0.8 | 18.1 | 2.3 |
|  | 128 | Pre | 0.3 | 0.0 | 0.0 | 0.3 | 5.9 | 0.2 |
|  |  | Post | 0.2 | 0.0 | 0.0 | 0.3 | 5.4 | 0.5 |
| *Tallaperla spp.* | 2 | Pre | 0.3 | 0.0 | 0.1 | 0.1 | 1.4 | 0.0 |
|  |  | Post | 0.3 | 0.0 | 0.0 | 0.2 | 3.8 | 0.2 |
|  | 8 | Pre | 0.6 | 0.0 | 0.0 | 0.3 | 5.9 | 1.4 |
|  |  | Post | 0.3 | 0.0 | 0.1 | 0.2 | 1.8 | 2.5 |
|  | 16 | Pre | 0.3 | 0.0 | 0.0 | 0.2 | 2.5 | 0.1 |
|  |  | Post | 0.2 | 0.0 | 0.1 | 0.3 | 7.0 | 1.4 |
|  | 32 | Pre | 0.1 | 0.0 | 0.0 | 0.2 | 1.8 | 0.2 |
|  |  | Post | 0.6 | 0.0 | 0.0 | 0.5 | 8.4 | 1.3 |
|  | 128 | Pre | 0.1 | 0.0 | 0.0 | 0.1 | 1.4 | 0.0 |
|  |  | Post | 0.2 | 0.0 | 0.1 | 0.2 | 2.8 | 0.0 |

**Supplementary Table 2.** Elemental limits of detection (LOD). R^2^-values ranged from 0.9997-1 across all elements, and % recovery of internal yttrium standard ranged from 92-100%. All sample measurements were automatically corrected for changes in % recovery over the course of the run

| Element | LOD (μg L^-1^) |
| --- | --- |
| Al | 4 |
| Ba | 0.2 |
| Ca | 7 |
| Cd | 0.1 |
| Co | 0.2 |
| Cu | 2.0 |
| Fe | 2 |
| K | 1 |
| Li | 0.1 |
| Mg | 0.4 |
| Mn | 0.1 |
| Na | 0.3 |
| P | 17 |
| S | 14 |
| Si | 1 |
| Sr | 0.1 |
| Zn | 0.5 |

| Genus | CBOM NP | Treatment | Consumer Production | Consumer Production | Mean Production |
| --- | --- | --- | --- | --- | --- |
|  | (mol) |  | (mg AFDM m^-2^ yr^-1^) | (lnRR) | (lnRR) |
| *Maccaffertium* | 52 | Pre | 16 | 0.63 | 0.70 |
|  | 43 | Enr | 30 |  |  |
|  | 49 | Pre | 16 | 0.12 |  |
|  | 46 | Enr | 19 |  |  |
|  | 50 | Pre | 21 | 1.03 |  |
|  | 38 | Enr | 58 |  |  |
|  | 52 | Pre | 2 | 1.42 |  |
|  | 39 | Enr | 7 |  |  |
|  | 50 | Pre | 19 | 0.28 |  |
|  | 37 | Enr | 25 |  |  |
| *Pycnopsyche* | 52 | Pre | 112 | 0.58 | 0.54 |
|  | 43 | Enr | 200 |  |  |
|  | 49 | Pre | 66 | -0.05 |  |
|  | 46 | Enr | 63 |  |  |
|  | 50 | Pre | 58 | 0.46 |  |
|  | 38 | Enr | 92 |  |  |
|  | 52 | Pre | 64 | 1.21 |  |
|  | 39 | Enr | 214 |  |  |
|  | 50 | Pre | 53 | 0.49 |  |
|  | 37 | Enr | 87 |  |  |
| *Tallaperla* | 52 | Pre | 35 | 1.15 | 0.70 |
|  | 43 | Enr | 109 |  |  |
|  | 49 | Pre | 19 | 0.74 |  |
|  | 46 | Enr | 40 |  |  |
|  | 50 | Pre | 34 | 1.22 |  |
|  | 38 | Enr | 115 |  |  |
|  | 52 | Pre | 20 | 0.79 |  |
|  | 39 | Enr | 44 |  |  |
|  | 50 | Pre | 77 | -0.40 |  |
|  | 37 | Enr | 51 |  |  |

**Supplementary Table 3.** Differences in secondary production responses to nutrient enrichment. Consumer responses are presented as natural log response ratios (lnRR) calculated by dividing the natural log (ln) of enriched production estimates (Enr) by ln pre-enrichment (Pre) production estimates. Consumer production values are given as ash free dry mass (AFDM), and enrichment values are reported for coarse benthic organic matter nitrogen:phosphorus ratios (CBOM N:P)
